# Four new species of the genus *Saigona* Matsumura (Hemiptera, Fulgoromorpha, Dictyopharidae) from China

**DOI:** 10.3897/zookeys.462.7500

**Published:** 2014-12-10

**Authors:** Yan-Li Zheng, Lin Yang, Xiang-Sheng Chen

**Affiliations:** 1 The Provincial Key Laboratory for Agricultural Pest Management of Mountainous Region; 2 Institute of Entomology, Guizhou University, Guizhou Province, 550025 China 2 Guizhou Normal College, Guizhou Province, 550018 China

**Keywords:** Fulgoroidea, Oriental region, Palaearctic region, planthopper, taxonomy

## Abstract

Four new species of the genus *Saigona* Matsumura, 1910, *Saigona anisomorpha* Zheng, Yang & Chen, **sp. n.**, *Saigona daozhenensis* Zheng, Yang & Chen, **sp. n.**, *Saigona dicondylica* Zheng, Yang & Chen, **sp. n.** and *Saigona tenuisa* Zheng, Yang & Chen, **sp. n.**, from China, are described and illustrated. A key to the species of *Saigona* is provided.

## Introduction

The dictyopharid planthopper genus *Saigona* was established by Matsumura (1910) for Dictyophora [sic] ishidae Matsumura, 1905, from Japan. Recently, Liang and Song (2006) revised this genus and recognized the following 9 valid species: *Saigona capitata* (Distant, 1914) (Indo-China, S.W. China: Yunnan), *Saigona fulgoroides* (Walker, 1858) (S. China, Sumatra, Borneo), *Saigona fuscoclypeata* Liang & Song, 2006 (C. China: Shaanxi, Hubei and Gansu), *Saigona henanensis* Liang & Song, 2006 (C. China: Henan), *Saigona latifasciata* Liang & Song, 2006 (S.W. China: Yunnan), *Saigona robusta* Liang & Song, 2006 (C. China: Hubei), *Saigona sinicola* Liang & Song, 2006 (C. China: Shaanxi), *Saigona taiwanella* Matsumura, 1941 (China: Taiwan), and *Saigona ussuriensis* (Lethierry, 1878) (Russian: Far Eastern Region, Japan, Korea, N.E. China: Jilin, Heilongjiang) (Liang and Song 2006). Subsequently, Zheng and Chen (2011) added a new species, *Saigona saccus* Zheng, Yang & Chen, 2011 from Guizhou Province, China.

While sorting and identifying Dictyopharidae from material in the Institute of Entomology, Guizhou University (IEGU), we found four new species of *Saigona*, which are herein described and illustrated. The purpose of this paper is to describe these four new species and to provide an identification key to the species of this genus.

## Material and methods

The morphological terminology and measurements used in this study follow Liang and Song (2006). The genital segments of the examined specimens were macerated in 10% NaOH and drawn from preparations in glycerin using a light microscope. Figures of the specimens were made using Leica MZ12.5. Spinal formula of hind leg means the numbers of spines of the tibia, the lateral spines spread along the lateral margin, plus the 1^st^ and 2^nd^ tarsomeres.

The following abbreviations are used in the text, BL: body length (from apex of cephalic process to tip of fore wings); HL: head length (from apex of cephalic process to base of eyes); HW: head width (including eyes); FWL: forewing length.

The type specimens are deposited in the Institute of Entomology, Guizhou University, China (IEGU).

## Taxonomy

### Genus Saigona

Taxon classificationAnimaliaHemipteraDictyopharidae

Matsumura, 1910

Saigona Matsumura, 1910: 110; Melichar 1912: 28, 50; Metcalf 1946: 47; Nast 1972: 84; Chou et al. 1985: 63; Anufriev and Emeljanov 1988: 482; Emeljanov 1993: 70; Liang 2001: 235. Type species: Dictyophora [sic] ishidae Matsumura, 1905 [=*Almana ussuriensis* Lethierry, 1878], by subsequent designation of Melichar 1912: 50; Liang and Song 2006: 28, by comprehensive redescription.
Leprota Melichar, 1912: 91; Metcalf 1946: 74. Type species: Dictyophora [sic] fulgoroides Walker, 1858, by original designation and monotypy. [Synonymised by Liang and Song 2006: 28.]
Neoputala Distant, 1914: 412; Metcalf 1946: 78. Type species: *Neoputala lewisi* Distant, 1906 [not *Neoputala capitata* Distant, 1914, as stated by Liang 2001: 236]. [Synonymised by Liang 2001: 236.]
Piela Lallemand, 1942: 72. Type species: *Piela singularis* Lallemand, 1942, by original designation and monotypy. [Synonymised by Liang and Song 2006: 28.]

#### Type species.

Dictyophora [sic] ishidae Matsumura, 1905 (original designation).

#### Diagnosis.

For the relationships and diagnosis of *Saigona* see Liang and Song (2006: 28).

#### Distribution.

China (Yunnan, Guizhou, Guangxi, Guangdong, Sichuan, Hubei, Hunan, Jiangxi, Zhejiang, Fujian, Gansu, Shaanxi, Henan, Taiwan, Jilin, Heilongjiang); Korea; Indochina; Japan; Russia (Far Eastern Region).

#### Key to species of the genus *Saigona* Matsumura

(Modified from Liang and Song 2006 and updated five species)

**Table:** 

1	Vertex with cephalic process short, shorter than pronotum and mesonotum combined (Figs 1, 23)	**2**
–	Vertex with cephalic process long, longer than or nearly as long as pronotum and mesonotum combined (Figs 12, 34)	**7**
2	Postclypeus yellowish or yellowish brown	**3**
–	Postclypeus fuscous	**5**
3	Mesonotum with a yellowish stripe along median longitudinal carina	**4**
–	Mesonotum without a yellowish stripe along median longitudinal carina (Fig. 51)	***Saigona dicondylica* sp. n.**
4	Mesonotum with median longitudinal yellowish stripe narrower; aedeagus with phallobase with apical ventral membranous lobe with numerous, fine spines at apex	***Saigona ussuriensis* (Lethierry)**
–	Mesonotum with median longitudinal yellowish stripe much broader; aedeagus with phallobase with apical dorsal and ventral membranous lobes with numerous, fine spines at apex	***Saigona latifasciata* Liang & Song**
5	Frons with lateral carinae not reaching to frontoclypeal suture	***Saigona fuscoclypeata* Liang & Song**
–	Frons with lateral carinae almost reaching to frontoclypeal suture (Fig. 2)	**6**
6	Aedeagus with phallobasal conjunctival processes spiraled dorsally	***Saigona henanensis* Liang & Song**
–	Aedeagus with phallobasal conjunctival processes not spiraled dorsally (Figs 9–11)	***Saigona anisomorpha* sp. n.**
7	Cephalic process bulbous apically, with 3 pairs of symmetrical knoblike protuberance on lateral regions	**8**
–	Cephalic process not bulbous apically, without knoblike protuberance on lateral regions	**9**
8	Aedeagus with phallobasal conjunctival processes straight	***Saigona saccus* Zheng, Yang & Chen**
–	Aedeagus with phallobasal conjunctival processes reflexed laterad at apex	***Saigona fulgoroides* (Walker)**
9	Frons with lateral carinae almost reaching to frontoclypeal suture	***Saigona capitata* (Distant)**
–	Frons with lateral carinae reaching to eyes, not to frontoclypeal suture (Figs 13, 35)	**10**
10	Mesonotum with yellowish stripe along median longitudinal carina very narrow	***Saigona taiwanella* Matsumura**
–	Mesonotum with yellowish stripe along median longitudinal carina broad (Figs 12, 48, 49)	**11**
11	Posterior margin of pygofer produced into a large process dorsally (Fig. 40)	***Saigona tenuisa* sp. n.**
–	Posterior margin of pygofer not produced into a large process dorsally	**12**
12	Aedeagus with phallobase with apical dorsal and ventral membranous lobes with numerous, fine spines at apex	***Saigona sinicola* Liang & Song**
–	Aedeagus with phallobase with apical ventral membranous lobe with numerous, fine spines at apex (Fig. 21)	**13**
13	Aedeagus with phallobasal conjunctival processes subparallel apically (Figs 20–22)	***Saigona daozhenensis* sp. n.**
–	Aedeagus with phallobasal conjunctival processes diverging apically	***Saigona robusta* Liang & Song**

**Figures 1–11.**
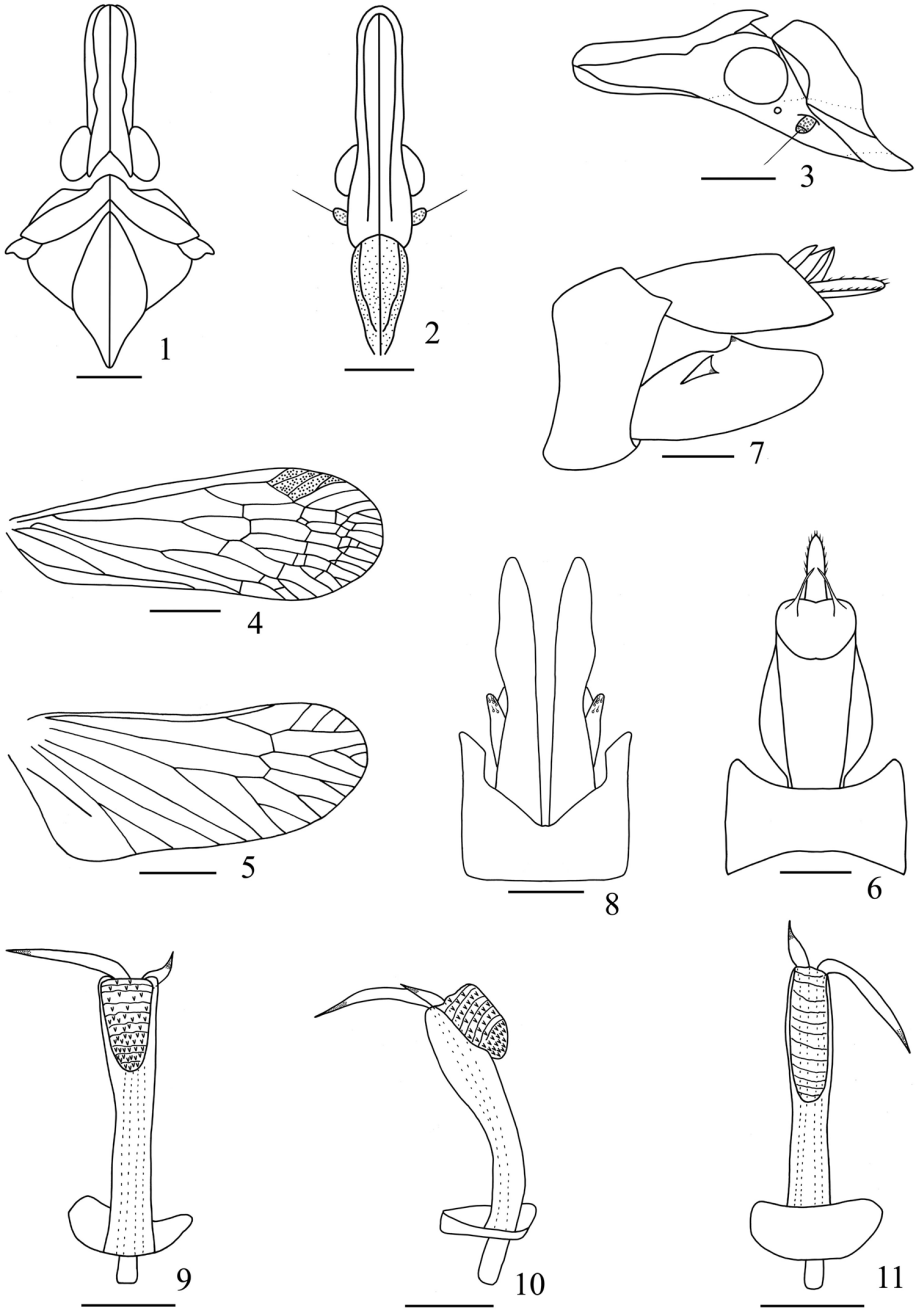
*Saigona anisomorpha* Zheng, Yang & Chen, sp. n. **1** Head and thorax, dorsal view **2** Frons and clypeus, ventral view **3** Head and pronotum, lateral view **4** Forewing **5** Hindwing **6** Pygofer and anal tube, dorsal view **7** Genitalia, lateral view **8** Pygofer and parameres, ventral view **9** Aedeagus, ventral view **10** Aedeagus, lateral view **11** Aedeagus, dorsal view. Scale bars: **1–5** = 1 mm, **6–11** = 0.5 mm.

**Figures 12–22.**
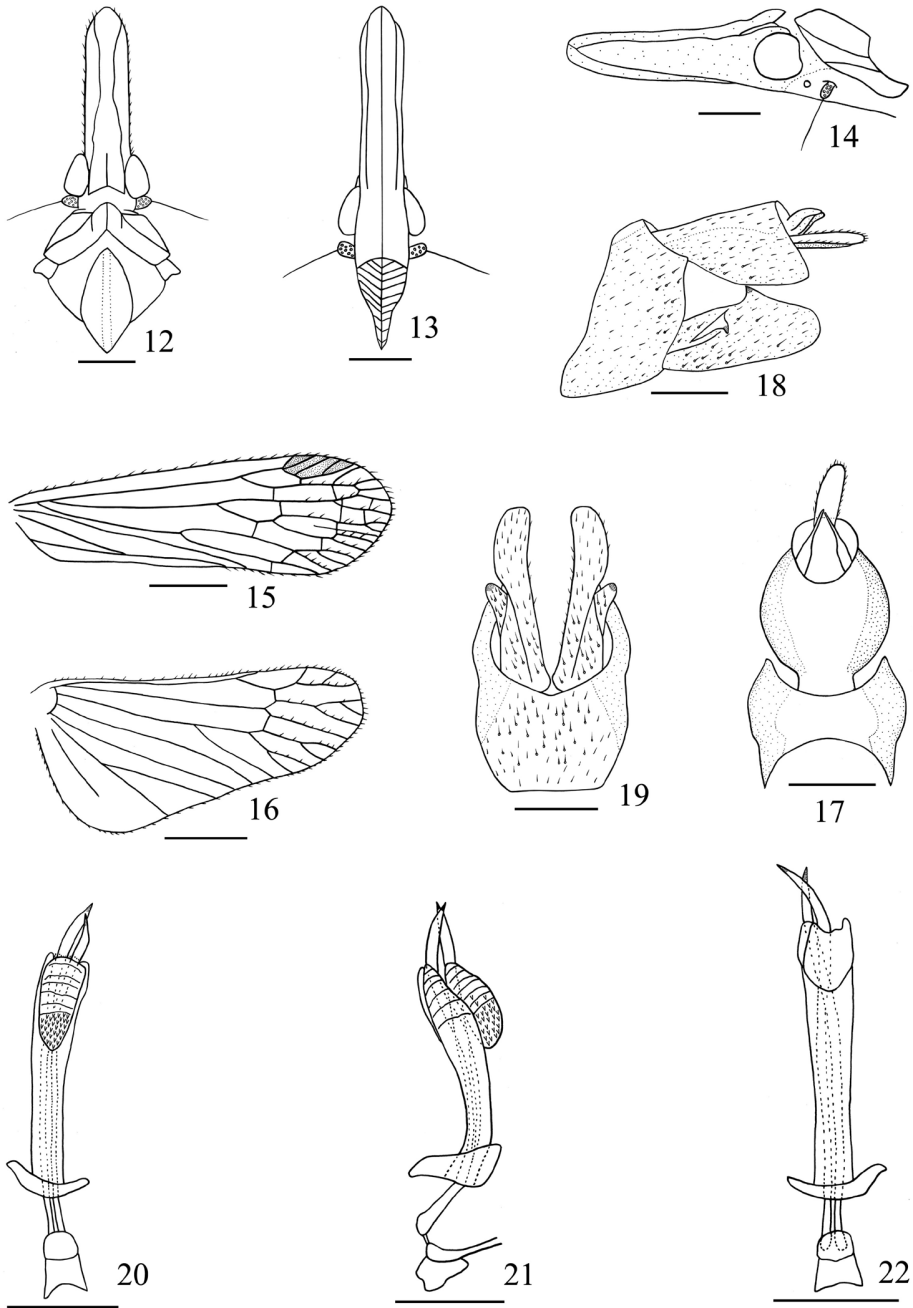
*Saigona daozhenensis* Zheng, Yang & Chen, sp. n. **12** Head and thorax, dorsal view **13** Frons and clypeus, ventral view **14** Head and pronotum, lateral view **15** Forewing **16** Hindwing **17** Pygofer and anal tube, dorsal view **18** Genitalia, lateral view **19** Pygofer and parameres, ventral view **20** Aedeagus, ventral view **21** Aedeagus, lateral view **22** Aedeagus, dorsal view. Scale bars: **12–16** = 1 mm, **17–22** = 0.5 mm.

**Figures 23–33.**
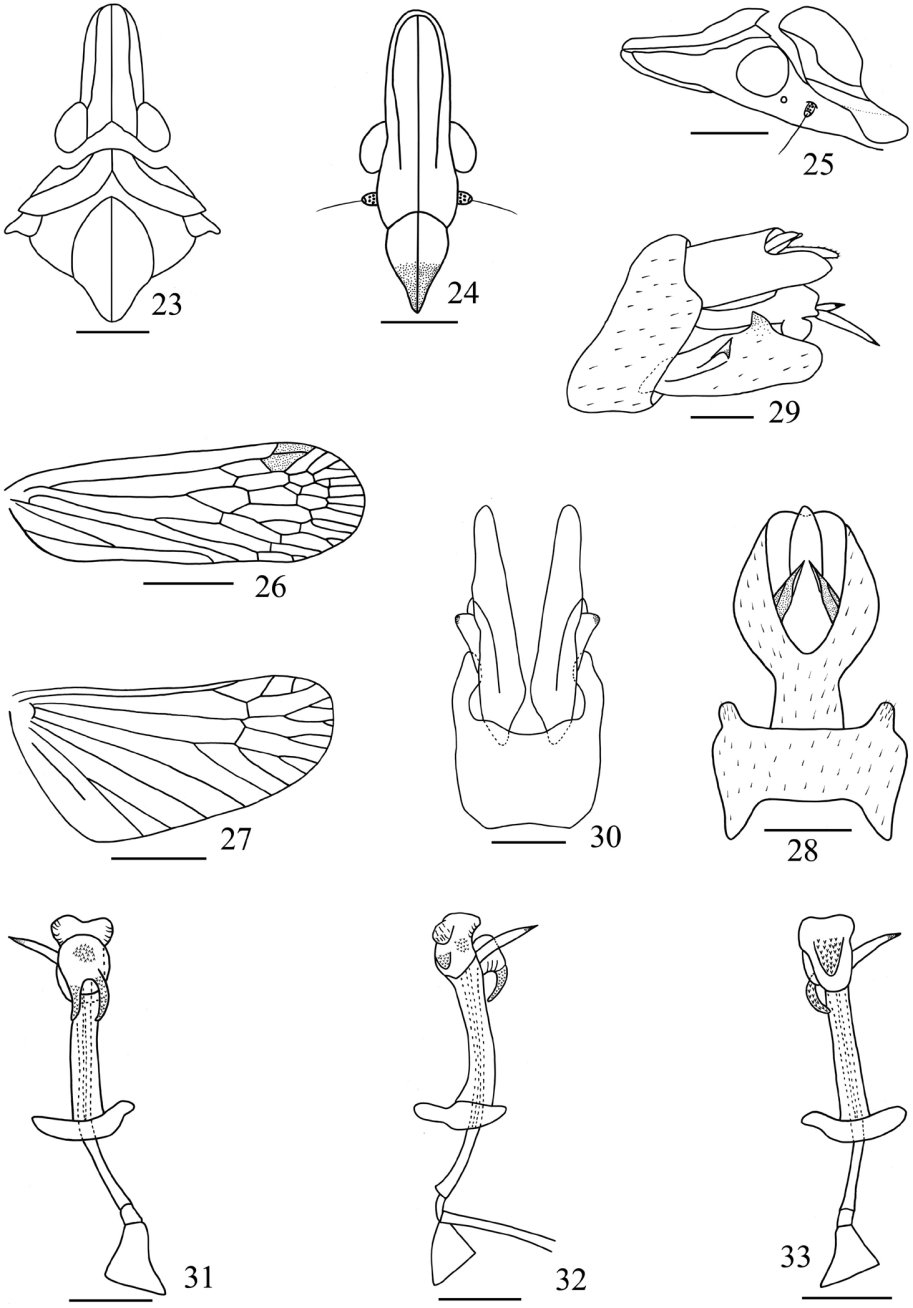
*Saigona dicondylica* Zheng, Yang & Chen, sp. n. **23** Head and thorax, dorsal view **24** Frons and clypeus, ventral view **25** Head and pronotum, lateral view **26** Forewing **27** Hindwing **28** Pygofer and anal tube, dorsal view **29** Genitalia, lateral view **30** Pygofer and parameres, ventral view **31** Aedeagus, ventral view **32** Aedeagus, lateral view **33** Aedeagus, dorsal view. Scale bars: **23–27** = 1 mm, **28–33** = 0.5 mm.

**Figures 34–44.**
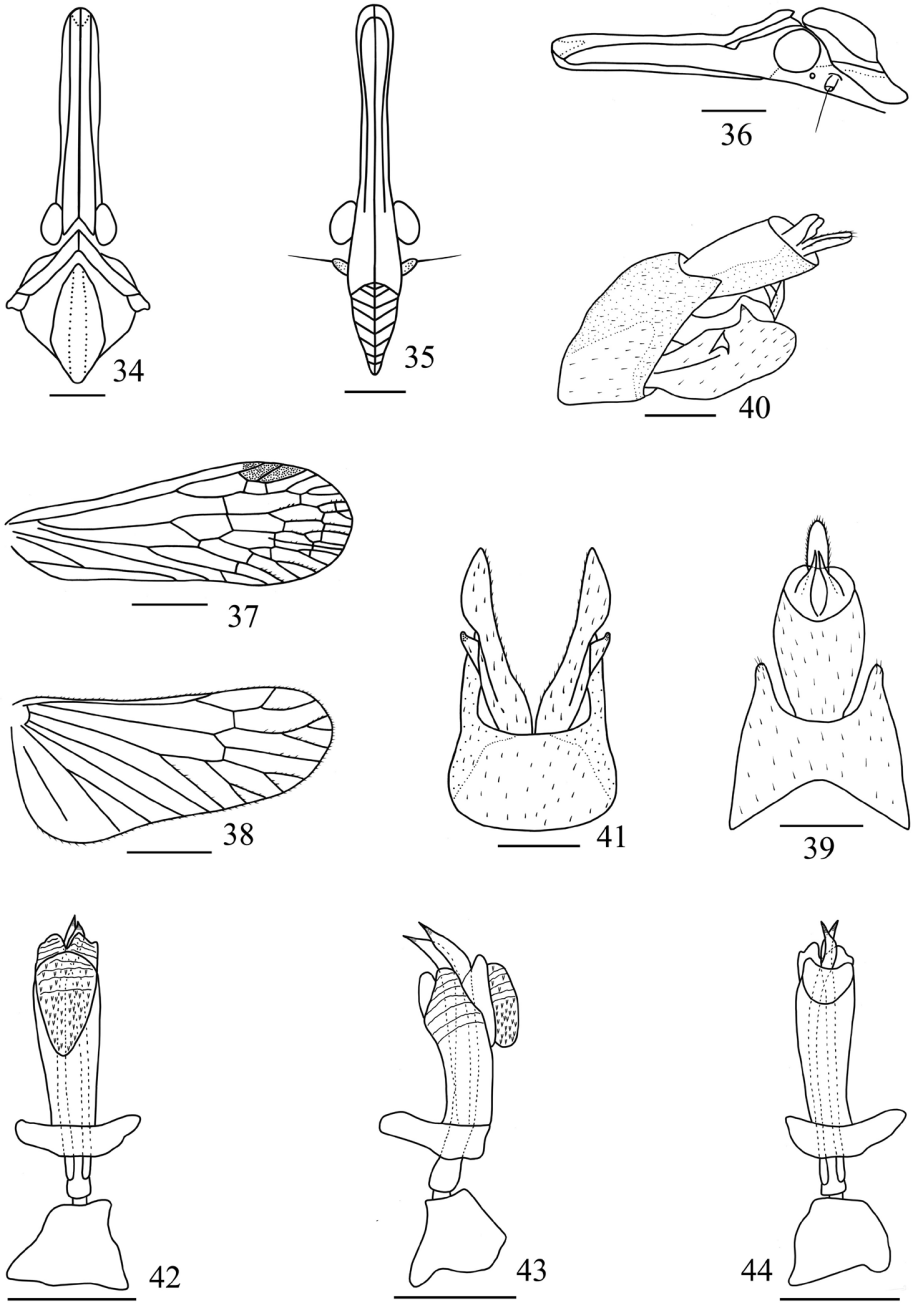
*Saigona tenuisa* Zheng, Yang & Chen, sp. n. **34** Head and thorax, dorsal view **35** Frons and clypeus, ventral view **36** Head and pronotum, lateral view **37** Forewing **38** Hindwing **39** Pygofer and anal tube, dorsal view **40** Genitalia, lateral view **41** Pygofer and parameres, ventral view **42** Aedeagus, ventral view **43** Aedeagus, lateral view **44** Aedeagus, dorsal view. Scale bars: **34–38** = 1 mm, **39–44** = 0.5 mm.

**Figures 45–50.**
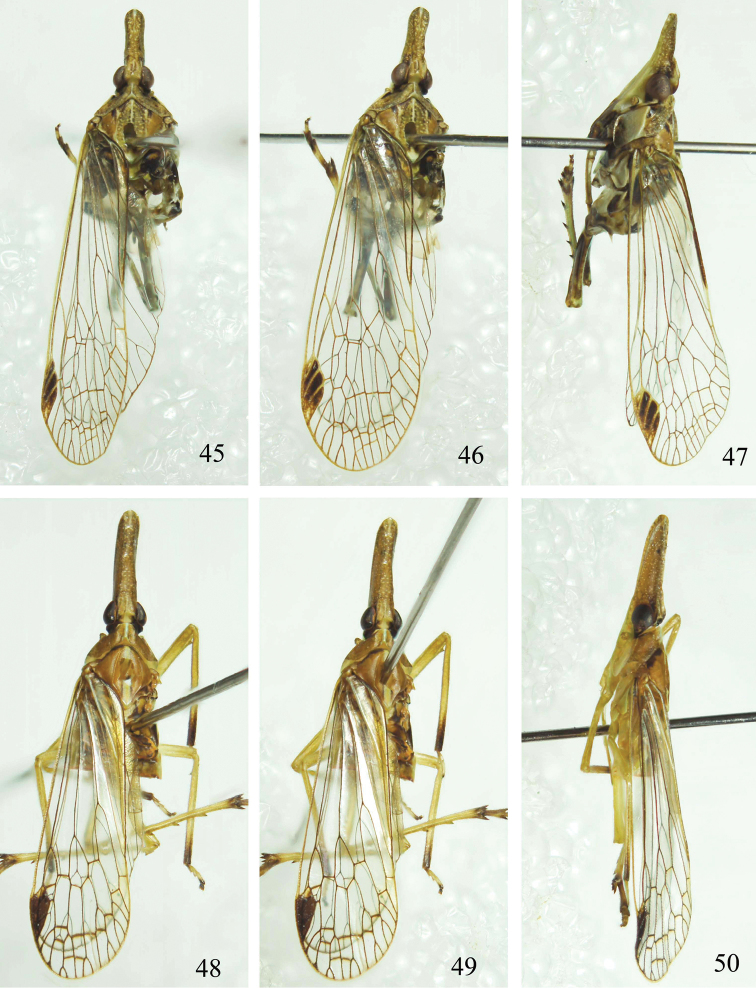
Habitus of *Saigona* species. **45–47** *Saigona anisomorpha* Zheng, Yang & Chen, sp. n.; **48–50** *Saigona daozhenensis* Zheng, Yang & Chen, sp. n. (**45, 48** dorsal view; **46, 49** dorsolateral view; **47, 50** lateral view).

**Figures 51–56.**
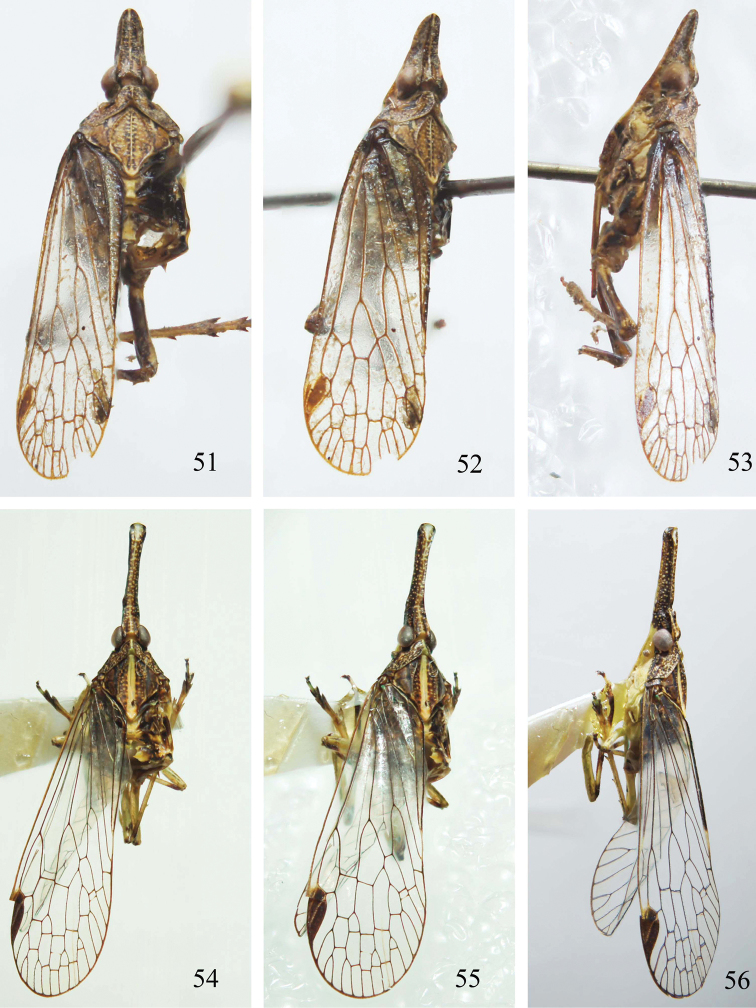
Habitus of *Saigona* species. **51–53** *Saigona dicondylica* Zheng, Yang & Chen, sp. n. **54–56** *Saigona tenuisa* Zheng, Yang & Chen, sp. n. (**51, 54** dorsal view; **52, 55** dorsolateral view; **53, 56** lateral view).

### Saigona anisomorpha

Taxon classificationAnimaliaHemipteraDictyopharidae

Zheng, Yang & Chen sp. n.

http://zoobank.org/9611A27A-2A7D-4B8C-ACD0-4CC0E2D9C744

Figs 1–11 , 45–47

#### Description.

*Measurement.* ♂, BL: 15.7 mm; HL: 2.4 mm; HW: 1.5 mm; FWL: 11.8 mm.

*Coloration.* General color brown, marked with fuscous and ochraceous (Figs 45–47). Vertex brown with median carina ochraceous, lateral margins brown. Genae brown, eyes brown, ocellus yellowish, antenna brown and the areas surrounding ocellus and antenna beneath eye yellowish. Frons yellowish. Postclypeus, anteclypeus black. Pronotum pale brown, lateral, ventrally curved areas with a yellowish band. Mesonotum pale brown scattered white spots and yellowish at the apex. Thorax ventrally at the fore femur brown, the other area pale green. Abdomen dorsally dark, with yellowish band on disc, ventrally dark. Forewings venation brown and stigma dark. Legs dark, tibiae with green rings. Genitalia black.

*Head and thorax.* Head (Figs 1, 45–47) shorter than pronotum and mesonotum combined (0.75:1). Vertex (Fig. 1) with median carina very faint, only conspicuous at apex and base; lateral carinate margins curved. Frons (Fig. 2) with lateral carinae almost reaching to to frontoclypeal suture. Mesonotum (Figs 1, 45–47) tricarinate on disc, lateral carinate curved towards media carinate at the front. Forewing (Figs 4, 45–47) longer than widest part (2.85:1), venation as in Fig. 4; hindwing longer than widest part (2.43:1), venation as in Fig. 5. Spinal formula of hind leg 8-12-11.

**Male genitalia.** Anal tube (Figs 6, 7) large, nearly triangular in lateral view (Fig. 41), large, long, round in dorsal view (Fig. 39), ratio of length to width at middle about 2:1. Pygofer (Fig. 7) large and broad in lateral view, posterior margin with a slightly sharp process dorsally. Parameres (Figs 7, 8) relatively large, broad in lateral aspect (Fig. 7), apex sharply rounded, protruded posteriorly. Aedeagus (Figs 9–11) with phallobasal conjunctival processes unequal in length, left one obviously longer than right one; phallobase narrow and long, curved dorsally; apical, dorsal, membranous lobe small in lateral view (Fig. 10), and long oval in dorsal view (Fig. 11); apical, ventral, membranous lobe converging towards apex and oval in ventral view (Fig. 9), directed anteroventrally in lateral view (Fig. 10), covered with numerous fine spines and veins.

#### Type material.

Holotype: ♂, **China:** Baiyun Mountain (N34°08', E112°05'), Henan Province, 13 Aug. 2008, X.-H. Hou. (IEGU).

#### Etymology.

This new species is named for its aedeagus with two phallobasal conjunctival processes unequal in length.

#### Distribution.

China (Henan).

#### Remarks.

This species is similar to *Saigona henanensis* Liang & Song, 2006, but can be distinguished from the latter by its phallobasal conjunctival processes not spiraled at apical 1/5, left one obviously longer than right one; phallobase with apical, dorsal small, with apical, ventral, membranous lobe small, not hook-like in lateral view.

### Saigona daozhenensis

Taxon classificationAnimaliaHemipteraDictyopharidae

Zheng, Yang & Chen sp. n.

http://zoobank.org/93A2289B-DD34-4A49-9F76-F307FE229E05

Figs 12–22 , 48–50

#### Description.

*Measurement.* ♂, BL: 15.8 mm; HL: 3.2 mm; HW: 1.5 mm; FWL: 10.5 mm.

*Coloration.* General color brown, marked with fuscous and ochraceous speckles (Figs 48–50). Vertex brown with lateral carinate balck, median carinate ochraceous. Genae brown, eyes brown, ocellus pink, antenna yellowish and the areas surrounding ocellus and antenna beneath eye yellowish. Frons yellowish, the apex of it black and media carina ochraceous. Pronotum brown with median carina yellowish; lateral, ventrally curved areas yellowish. Mesonotum ochraceous, with a narrow, yellow stripe along median longitudinal carina. Thorax ventrally yellowish; abdomen dorsally dark brown, with yellowish brown stripes, ventrally yellowish. Forewings with most veins fuscous, A and Cu yellowish, stigma dark brown. Legs pale yellowish, apex of tibia, digitus, claw pale brown. Pygofer, anal style and anal tube yellowish-brown.

*Head and thorax.* Head (Figs 12, 48–50) moderately long, longer than pronotum and mesonotum combined (1.18:1). Cephalic process relatively long and robust, somewhat upturned; Vertex (Fig. 12) with median carina very faint, only conspicuous at base, lateral carinate margins curved in front of eyes, disc conspicuous depressed. Frons (Fig. 32) with lateral carinate reaching to the front of eyes, not to frontoclypeal suture. Pronotum (Figs 1, 48–50) with median carina distinct, lateral carinae very faint; mesonotum with median longitudinal carina obsolete. Forewing (Figs 15, 48–50) longer than widest part (3.16:1), venations as in Fig. 15; hindwing longer than widest part (2.20:1), venations as in Fig. 16. Spinal formula of hind leg 8-12-10.

**Male genitalia.** Anal tube (Figs 17, 18) large, nearly triangular in lateral view; large, roundrf in dorsal view, ratio of length to width at middle about 1.2:1. Anal style (Figs 17, 18) short, broad. Pygofer (Fig. 18) in lateral view with posterior margin slightly sinuate. Parameres (Figs 18, 19) with one robust spines laterally. Aedeagus (Figs 20–22) with phallobasal conjunctival processes produced posteriorly, asymmetry; phallobase narrow and long, curved dorsally; apical, dorsal, membranous lobe small in lateral view (Fig. 21), without spines; apical, ventral, membranous lobe converging towards apex and semi-oval in ventral view (Fig. 20), directed anteroventrally in lateral view (Fig. 21), covered with numerous fine spines at apex.

#### Type material.

Holotype: ♂, **China:** Sanqiao Town (N28°53', E107°36', 1,300–1,600 m), Daozhen County, Guizhou Province, 22–24 May 2004, X.-S. Chen. (IEGU).

#### Etymology.

The specific name refers to the locality, Daozhen County, Guizhou Province, China.

#### Distribution.

China (Guizhou).

#### Remarks.

This species is similar to *Saigona robusta* Liang & Song, 2006, but can be distinguished from the latter by its anal tube rounded in dorsal view (oval in *robusta*); phallobasal conjunctival processes produced posteriorly (produced dorsally and ventrally, respectively in *robusta*); phallobase with apical, ventral, membranous lobe small, semi-oval in ventral view (large, triangular in *robusta*).

### Saigona dicondylica

Taxon classificationAnimaliaHemipteraDictyopharidae

Zheng, Yang & Chen sp. n.

http://zoobank.org/B185FB7C-239B-4191-8D0A-288F6936ED75

Figs 23–33 , 51–53

#### Description.

*Measurement.* ♂, BL: 12.0 mm; HL: 1.9 mm; HW: 1.4 mm; FWL: 8.5 mm.

*Coloration.* General color brown, marked with fuscous and ochraceous (Figs 51–53). Vertex dark scattered numerous yellowish spots, median carina ochraceous, lateral carinae and margin dark. Genae brown, eyes brown, ocellus yellowish, antenna dark and the areas surrounding ocellus and antenna beneath eye brown scattered yellowish spots. Frons pale brown with brown spots. Postclypeus, anteclypeus yellowish-brown and the apex black. Pronotum dark scattered brown spots, median carina brown, lateral carinae dark; lateral, ventrally curved areas brown. Mesonotum ochraceous scattered black brown spots. Thorax ventrally dark brown. Forewings with venation and stigma brown. Legs dark with pale brown spots.

*Head and thorax.* Head (Figs 23–25, 51–53) slightly short and robust, shorter than pronotum and mesonotum combined (0.67:1). Vertex (Figs 23, 51, 52) with conspicuous median carina; disc sunking distinct. Frons (Fig. 24) with lateral carinae reaching to the behind of eyes, not to frontoclypeal suture. Pronotum (Figs 23, 51, 52) with distinct median carina, lateral carinae curved. Mesonotum (Fig. 23) tricarinate on disc, lateral carinae curved towards median carinae at the front. Forewing (Figs 26, 51–53) longer than widest part (3.06:1), venations as in Fig. 26; hindwing longer than widest part (2.13:1), venations as in Fig. 27. Spinal formula of hind leg 8-11-11.

**Male genitalia.** Anal style (Figs 28, 29) short, broad. Anal tube (Figs 28, 29) large, nearly oval in lateral view; long, capitate in dorsal view, ratio of length to width at middle about 1.5:1. Pygofer (Figs 28–30) large and broad in lateral view, posterior margin with a blunt process dorsally. Parameres (Figs 29, 30) relatively long in ventral aspect. Aedeagus (Figs 31–33) with phallobasal conjunctival processes produced dorsally and ventrally, respectively; left one obviously longer than right one; phallobase narrow and long, curved dorsally; apicodorsal membranous lobe large in lateral view (Fig. 32), with two small processes, one covered with numerous fine spines, another not; apicoventral membranous lobe converging towards apex and rounded in ventral view (Fig. 31) with two stout spine-like processes, directed anteroventrally in lateral view, covered with numerous fine spines.

#### Type material.

Holotype: ♂, **China:** Yujun Mountain (N30°04', E101°57', 3,000 m), Kangding County, Sichuan Province, 6 Aug. 1999, G.-D. Ren. (IEGU).

#### Etymology.

This new species is named for its aedeagus having two stout spine-like processes apically.

#### Distribution.

China (Sichuan).

#### Remarks.

This species is similar to *Saigona ussuriensis* (Lethierry, 1878), but can be distinguished from the latter by its anal tube obviously narrowing at basal third; apicoventral lobe of pallobase with two stout processes at apex; phallobasal conjunctival processes asymmetrical apically.

### Saigona tenuisa

Taxon classificationAnimaliaHemipteraDictyopharidae

Zheng, Yang & Chen sp. n.

http://zoobank.org/35594B4E-E88C-4967-890B-DCBE0E61786F

Figs 34–44 , 54–56

#### Description.

*Measurement.* ♂, BL: 15.8 mm; HL: 3.9 mm; HW: 1.4 mm; FWL: 9.2 mm.

*Coloration.* General color dark brown (Figs 54–56). Vertex dark brown marked with fuscous and ochraceous and yellowish spots at apex. Genae pale brown, eyes pale brown, ocellus yellowish, antenna green and the areas surrounding ocellus and antenna beneath eye yellowish. Frons yellowish brown. Postclypeus, anteclypeus and rostrum yellowish. Pronotum and Mesonotum dark brown scattered yellowish spots. Pronotum with median carina yellowish green, lateral, ventrally curved areas yellowish. Mesonotum with a narrow, yellow stripe along median carina. Thorax ventrally yellowish; abdomen ventrally yellowish green, dorsally black with yellowish brown stripe on median carina. Forewings with most veins and stigma dark brown. Legs yellowish green, tibiae with brown ring spots. Genitalia black.

*Head and thorax.* Head (Figs 34–36, 54–56) very long, longer than pronotum and mesonotum combined (1.36:1). Vertex (Figs 34, 54–56) with median carina complete; cephalic process long and slender, somewhat upturned. Frons (Fig. 35) with lateral carinae reaching to the front of eyes, not to frontoclypeal suture. Pronotum (Fig. 34) with median carina distinct, lateral carinae very faint. Mesonotum (Fig. 34) with median longitudinal carina obsolete or unconspicuous, lateral carinae curverging anteriorly. Forewing (Figs 37, 54–56) longer than widest part (2.79:1), venations as in Fig. 37; hindwings longer than widest part (2.23:1), venations as in Fig. 38. Spinal formula of hind leg 8-11-11.

**Male genitalia.** Anal style (Figs 39, 40) short, broad. Anal tube (Figs 39, 40) large, nearly triangular in lateral view; long, oval in dorsal view, ratio of length to width at middle about 1.7:1. Pygofer (Figs 39–41) in lateral view with posterior margin slightly concave, dorsoposterior angle produced into a sharply process. Aedeagus (Figs 42–44) with phallobasal conjunctival processes slightly produced dorsally, symmetrical; phallobase narrow and long, curved dorsally; apicodorsal membranous lobe small in lateral view (Fig. 43); apicoventral membranous lobe large, converging towards apex and coniform in ventral view (Fig. 42), directed anteroventrally in lateral view (Fig. 43), covered with numerous fine spines.

#### Type material.

Holotype: ♂, **China:** Forest Park (N26°35', E106°42'), Guiyang City, Guizhou Province, 19 July 2000, X.-S. Chen. (IEGU).

#### Etymology.

The species name is derived from the Hellenic word “*tenuisa*”, referring to cephalic process slender and long.

#### Distribution.

China (Guizhou).

#### Remarks.

This species can be easily distinguished from other species of *Saigona* by its very long, slender cephalic process.

## Supplementary Material

XML Treatment for Saigona

XML Treatment for Saigona anisomorpha

XML Treatment for Saigona daozhenensis

XML Treatment for Saigona dicondylica

XML Treatment for Saigona tenuisa
